# Daily fluctuations in adolescents' sleep predict next‐day attention, sleepiness, and fatigue: an ecological momentary assessment study over 28 days

**DOI:** 10.1111/jcpp.14076

**Published:** 2024-12-01

**Authors:** Lin Shen, Jessica Nicolazzo, Tracey L. Sletten, Clare Anderson, Yang Yap, Joshua F. Wiley, Bei Bei

**Affiliations:** ^1^ Victorian Catholic Education Authority Melbourne Vic. Australia; ^2^ Turner Institute for Brain and Mental Health, School of Psychological Sciences, Faculty of Medicine, Nursing and Health Sciences Monash University Clayton Vic. Australia; ^3^ School of Psychology University of Birmingham Birmingham UK; ^4^ School of Health and Biomedical Sciences RMIT University Melbourne Vic. Australia; ^5^ Peter MacCallum Cancer Centre Melbourne Vic. Australia; ^6^ Women's Mental Health Service, Royal Women's Hospital, Department of Psychiatry University of Melbourne Melbourne Vic. Australia

**Keywords:** Adolescent, sleep duration, sleep efficiency, alertness, psychomotor vigilance task, sleepiness, fatigue, ecological momentary assessment

## Abstract

**Background:**

Current understanding of the associations between adolescents' daily sleep and daytime alertness and fatigue under naturalistically occurring restricted (school) and unrestricted (vacation) sleep opportunities is limited.

**Methods:**

A convenience sample of adolescents (*n* = 205; 54.1% females, *M*_age_ ± *SD* = 16.9 ± 0.87 years) completed daily measures of sleep, alertness, and fatigue over 28 days (2 weeks during school, and the subsequent 2‐week vacation). Actigraphy and sleep diary total sleep time (TST) and sleep efficiency (SE) were measured. Participants self‐reported sleepiness and fatigue every morning and afternoon, and completed a tablet‐based, 3.2‐min psychomotor vigilance task (PVT) every afternoon. Cross‐lagged multilevel models tested daily TST and SE as predictors of next‐day subjective sleepiness/fatigue and PVT performance. Between‐ (i.e., differences between individuals) and within‐person associations (i.e., whether nights with higher‐than‐individual's‐average TST/SE, predict next‐day outcomes) were tested simultaneously. Covariates included previous‐day outcome, day of the week, study day (1–28), school/vacation, chronotype, and sociodemographic variables.

**Results:**

Within‐persons, higher‐than‐average TST and SE (both actigraphy and diary) predicted better next‐day PVT performance (all *p* ≤ .006), and lower subjective sleepiness and fatigue the following morning and afternoon (all *p* ≤ .032). Between‐persons, adolescents with higher overall diary SE had lower morning subjective sleepiness (*p* < .001) and fewer PVT false starts in the afternoon (*p* = 0.02).

**Conclusions:**

Nights with longer‐ and higher‐than‐average sleep efficiency (both actigraphy and diary) predicted better daytime alertness and fatigue, both when examined objectively via sustained attention and via self‐report. These findings are relevant for understanding the significance of sleep for adolescents' day‐to‐day alertness levels and fatigue, particularly in the context of classroom learning and road safety.

## Introduction

Adolescence is a critical life stage for sleep–wake regulation. Maturational delays in circadian timing and reductions in homeostatic sleep pressure (Carskadon, 2011), increasing academic demands (Gillen‐O'Neel, Huynh, & Fuligni, 2013), increased prebedtime electronic usage, and reduced parental control over bedtime (Bryant & Gómez, 2015) all contribute to later sleep timings in adolescence. As early school start times dictate early wake times on school days, most adolescents obtain less than the recommended 8–10 hrs of sleep on school nights (Hirshkowitz et al., 2015; Paruthi et al., 2016). Thus, it is not surprising that 20%–50% of adolescents report daytime sleepiness, fatigue, and difficulty concentrating (Ghandour, Overpeck, Huang, Kogan, & Scheidt, 2004; Rimes et al., 2007; Roberts, Roberts, & Chen, 2002; ter Wolbeek, van Doornen, Kavelaars, & Heijnen, 2006).

Short and poor sleep are consistently associated with poorer alertness and higher fatigue across different measures, including self‐reported sleepiness and fatigue (Anderson, Storfer‐Isser, Taylor, Rosen, & Redline, 2009; Kerkhof, 2017; Short, Gradisar, Lack, Wright, & Dohnt, 2013), sustained attention, and working memory (DiFrancesco, Van Dyk, Altaye, Drummond, & Beebe, 2019; Lo, Ong, Leong, Gooley, & Chee, 2016; Louca & Short, 2014; Sadeh, Gruber, & Raviv, 2003). Experimental evidence indicates that after 5 days of restricted sleep (simulated weekdays during school term), two nights of extended sleep (simulated weekend) was not sufficient for adolescents to fully recover from the cognitive impairments incurred across the simulated school week (Lo et al., 2016). However, whether these findings can translate to naturalistic conditions (i.e., school term vs. vacation) on a day‐to‐day basis remain unclear. For example, compared to prescribed sleep times in experimental conditions, adolescents may choose to sleep at different times depending on different psychosocial and contextual factors.

The current evidence of daytime alertness and fatigue is largely based on studies relying on between‐person differences (i.e., inter‐individual differences), such as averaging the variables over the study period (e.g., days or weeks). This approach does not consider the variable nature and within‐person changes of daily sleep and daytime functioning (Bei, Wiley, Trinder, & Manber, 2016). Several daily studies to date have highlighted the importance of examining sleep and functional outcomes at both the between‐ and within‐person levels. For example, a large multiwave study in adolescents (i.e., 2 weeks, repeated annually across 3 years; *N* = 750; aged 14–15) showed that irrespective of one's typical sleep duration, nights with shorter‐than‐average sleep consistently predicted higher fatigue the following day (Fuligni & Hardway, 2006). Similarly, in children (aged 8–11), higher self‐reported sleep quality predicted better working memory performance the following morning and afternoon (Könen, Dirk, & Schmiedek, 2015). They also noted that over the 4‐week observation, intraindividual variability (i.e., differences *within* individuals) was greater than interindividual variability (i.e., differences *between* individuals) for both sleep and cognitive functioning (Könen et al., 2015). These findings aptly demonstrate how these associations may be missed when only examining differences between, and not within, individuals. Nonetheless, whether these associations differ between school term versus vacation remain unclear.

### The current study

Previous studies with this sample have investigated daily associations between sleep and affect (Shen, Wiley, & Bei, 2021, 2022) and adolescents' daily sleep behavioral regulation (Maskevich, Shen, Drummond, & Bei, 2022). This study on the other hand investigated the daily associations between actigraphy and self‐reported sleep (duration and efficiency) with daytime alertness, fatigue, and objectively assessed daytime functioning over 28 consecutive days (2 weeks of school term, and the subsequent 2‐week vacation). Ecological momentary assessment (EMA) was used to examine adolescents' experiences in their natural environments, maximizing ecological validity and reducing potential recall bias (Shiffman, Stone, & Hufford, 2008). It was hypothesized that at the between‐person's level, longer sleep duration and higher sleep efficiency would be associated with higher attention and lower self‐reported levels of sleepiness and fatigue; at the within‐person's level, nights with longer and higher efficiency sleep compared to one' own, would be associated with better next‐day attention lower self‐reported sleepiness and fatigue. Given the known differences between adolescents' sleep during school terms versus vacations (Bei et al., 2014), this study also investigated whether school/vacation status played a moderating role, and it was hypothesized that these associations would be weaker over the vacation period.

## Methods

### Participants

Adolescents attending Years 10, 11, or 12 of high schools in Victoria, Australia, were invited to participate. Individuals who attended the final 2 weeks of a school term, regularly used a smartphone with internet connection, and did not travel across time zones during the study, were eligible. To capture a community sample of adolescents, there were no exclusion criteria. A total of 205 adolescents completed the study (see Table 1 for descriptive profile).

**Table 1:** Sociodemographic characteristics of the sample (*N* = 205)

Participant characteristics	*M* (*SD*)/*n* (%)
Age, *M* (*SD*)	16.9 (0.9)
Female, *N* (%)	111 (54.1)
Body Mass Index, *M* (*SD*)	21.9 (4.4)
School year level, *n* (%)	
Year 10	51 (24.9)
Year 11	82 (40.0)
Year 12	72 (35.1)
Ethnicity, *n* (%)	
South‐East Asian	24 (11.7)
North‐East Asian	37 (18.0)
Southern Asian	36 (17.6)
Mixed Asian	17 (8.3)
White/European	73 (35.6)
Other	18 (8.8)
Born in Australia, *n* (%)	136 (66.3)
Working part‐time or casual, *n* (%)	69 (33.7)
Parents married or de facto, *n* (%)	164 (80.0)
Participating in extracurricular activities, *n* (%)	134 (65.4)
Current depressive disorder, *n* (%)	9 (4.4)
Current anxiety disorder, *n* (%)	12 (5.9)
Current sleep disorder, *n* (%)	23 (11.2)
Morningness Eveningness Questionnaire	48.2 (7.7)
Days during vacation when sleep is affected by commitments, *n* (%)	6.1 (3.0)

### Design and procedures

This study followed the STROBE (Von Elm, Altman, Egger, & Pocock, 2007) and CREMAS (Stone & Shiffman, 2002) guidelines for reporting of observational and EMA studies. Data collection occurred during each of the seven school terms between November 2017 and July 2019. Each adolescent participated in one of the seven data collection rounds, with each round lasting 28 consecutive days: last 2 weeks of a school term, and 2 weeks of the subsequent vacation. Adolescents were recruited via online advertisements, printed posters, and in‐person recruitment in public areas (e.g., shopping malls). Participants' parents or legal guardians provided informed consent online, and participants provided informed assent online, which was subsequently confirmed via telephone. Participants also attended an in‐person induction session, where a researcher familiarized them with all study equipment and procedures (e.g., daily surveys), including a practice round of the performance vigilance task (PVT).

Demographic information was collected at baseline via an online survey; chronotype was measured once at study entry in a school week, and once more in the second week of the vacation. During the 28‐day study, participants wore a wrist‐worn MiniMitter Actiwatch (models –L, –64, –2, Spectrum, Pro and Plus; Mini Mitter, Respironics, Bend, OR, USA) and completed daily surveys every morning and afternoon via a smartphone application (MetricWire Inc.). A sleep diary was completed as part of the morning survey, and sleepiness and fatigue were self‐reported every morning and afternoon. The PVT (Joggle Research Inc., Seattle, WA, USA) was completed via an iPad distributed to each participant. Participants were instructed to complete the PVT before the afternoon survey, as the survey contained questions on distraction, effort, and motivation when completing the cognitive tasks. The morning survey and afternoon tasks (PVT and survey) were open 7:00 a.m.–2:30 p.m., and 3:30 p.m.–7:00 p.m., respectively. These time windows were set to be outside of typical school hours, and the morning survey extended into the early afternoon in consideration of possible late rise‐times on nonschool days. Within these time windows, hourly on‐screen mobile notification reminders were sent until either the relevant survey was completed, or the time window ended, whichever was earlier. Participants were instructed to complete the morning survey as soon as they woke and the afternoon tasks as soon as possible after school ended. On average, the morning survey was completed within an hour of self‐reported rise times.

Participants were reimbursed up to $76 AUD for completing the study. To balance effort and motivation for the daily tasks, the reimbursement was based on $1 per day for completing all daily tasks (i.e., surveys and cognitive tasks; up to $48), a $5 bonus for each week of complete surveys and cognitive tasks submissions, and a $1 per day bonus when the participant performed above average (compared to all other participants on that day) on all the cognitive tasks. All procedures were approved by Monash University's Human Research Ethics Committee (project ID: 10005).

### Measures

#### Sleep duration and efficiency

Total sleep time (TST) and sleep efficiency (SE) were derived from actigraphy and sleep diaries as measures of sleep duration and efficiency, respectively. Objective sleep was estimated using the wrist worn, MiniMitter Actiwatch, with the various models providing comparable estimates of activity counts and derived sleep estimates (Respironics, 2009). Data were collected in 60‐s epochs and analyzed using the Actiware software (v.6.0.9) on the ‘medium’ threshold. Participants used the event marker on the Actiwatch to indicate bedtime (getting into bed intending to sleep) and rise time (getting out of bed to start the day). Participants reported bedtime and rise time using items adapted from the Consensus Sleep Diary (Carney et al., 2012). To maximize the accuracy of bed‐ and rise time estimation, unified bed‐ and rise times, as well as time‐in‐bed were created based on all available data from actigraphy and sleep diaries (i.e., self‐reported bedtime and rise time, wrist activity, light, and event marker). Sleep diary sleep duration, as well as both sleep diary and actigraphy sleep efficiency were then calculated based on the unified bed‐ and rise times.

#### State sleepiness

State sleepiness was examined using a single‐item, 10‐point Karolinska Sleepiness Scale (KSS; Kaida et al., 2006) adapted from the original 9‐point scale (Åkerstedt & Gillberg, 1990). Participants reported their sleepiness in the past 10 min, on a scale of 1 (*Extremely alert*) to 10 (*Extremely sleepy, cannot stay awake*).

#### Daytime fatigue

Participants completed the Visual Analog of Fatigue Scale (VAFS; Monk, 1989), which asked them how fatigued they currently feel on a scale of 0–100, with higher scores indicating higher fatigue. This validated scale is widely used in field studies (Insana & Montgomery‐Downs, 2010).

#### Sustained attention

The PVT requires participants to maintain attention on the screen and respond as quickly as possible to an on‐screen stimulus (a millisecond timer) by tapping on the screen, while avoiding a premature response before the stimulus appears (i.e., a false start). This study used a validated, 3.2‐min PVT (Basner, Mollicone, & Dinges, 2011; Roach, Dawson, & Lamond, 2006) administered on an iPad (Joggle Research software, v2.4; Joggle Research Inc, Seattle, WA, USA). After each response to the stimulus, the millisecond counter stopped and the reaction time was displayed on the screen for 1 s. The interstimulus interval varied randomly from 2 to 5 s. As part of a larger study protocol, participants completed the PVT alongside other cognitive tasks (Visual Object Learning Task to assess visual learning and memory and Balloon Analog Risk Task to assess risk decision‐making). The PVT was always presented as the first task. The iPad screen was set to maximal brightness to ensure consistency across test administrations, and participants were instructed to sit upright when completing the task, and complete it in a quiet room without distractions such as noises and movements.

Sleep restriction, much like what most adolescents experience during the school term, is known to produce a systematic skew in the distribution of reaction times, toward the right (i.e., slower reaction times; Ratcliff & Van Dongen, 2011). The following five outcomes were derived: the reciprocal of the mean reaction time, the reciprocal of the mean reaction time for the slowest 10% of responses, the mean reaction time for the fastest 10% of responses, the number of lapses (defined as reaction times >355 ms; Basner et al., 2011), and the number of false starts (defined as reaction times <100 ms; Basner et al., 2011). In line with past studies (e.g., Drummond et al., 2005; Loh, Lamond, Dorrian, Roach, & Dawson, 2004; Thomann, Baumann, Landolt & Werth, 2014), the reciprocal of (a) mean reaction time and (b) mean reaction time for the slowest 10% of responses were used to decrease the contribution of long lapses and capture slowing of performance in the immediate and intermediate response domains (Dinges et al., 1997).

#### Covariates

The following covariates that can impact sleep and daytime alertness and fatigue were included in the fully adjusted multilevel models. Self‐reported covariates captured at baseline included school year (10, 11, and 12), biological sex (Male = 0, Female = 1), body mass index (BMI; calculated from self‐reported height and weight), ethnic/racial background (White (0)/nonWhite (1)), born in Australia (No = 0, Yes = 1), adolescents' current work status (not working = 0, working part‐time/casual = 1), parental marital status (married or de facto = 0, separated/divorced/widowed/prefer not to answer = 1), and chronotype [measured using the Morningness Eveningness Questionnaire (MEQ; Horne & Ostberg, 1976) with a continuous score ranging from 16 to 86 where scores of 41 and below indicate ‘evening types’, scores of 59 and above indicate ‘morning types’, and scores between 42 and 58 indicate ‘intermediate types’]. For the purpose as a covariate, we dichotomized race (see Table 1 for detailed breakdown of race/ethnicity). Daily covariates included study day (1–28), and day of the week. In the models with sleep predicting PVT outcomes, effort, motivation, and distraction were also included as covariates, as these are known to affect performance on cognitive tasks (Brose, Schmiedek, Lövdén & Lindenberger et al., 2012). Each item was answered on a scale of 1 (*Not at all motivated/ Not at all distracted/Did not put in any effort*) to 10 (*Extremely motivated/Extremely distracted/Put forth my best effort*).

### Statistical analyses

Cross‐lagged multilevel linear models tested actigraphy and sleep diary TST and SE as predictors of sleepiness, fatigue, and attention. Each of the five PVT variables and morning/afternoon self‐report sleepiness and fatigue were analyzed in separate models. As the number of PVT lapses and false starts are count variables, these outcome variables were modeled using multilevel Poisson models. Data cleaning and analyses were conducted in R (v4.2.0; R Core Team, 2022). Multilevel models were tested using the R packages *lme4* (v1.1‐27.1; Bates, Mächler, Bolker, & Walker, 2015) or *glmmADMB* (for multilevel Poisson models; v0.8.3.3; Fournier et al., 2012; Skaug, Fournier, Bolker, Magnusson, & Nielsen, 2016), and degrees of freedom and *p*‐values were estimated using *lmerTest* (v3.1‐3; Kuznetsova & Brockhoff, 2017). All models were estimated using restricted maximum likelihood (Singer & Willett, 2003), and tested at two‐tailed α = .05.

For each model, marginal and conditional *R* ^2^ were calculated. Marginal *R* ^2^ represents the proportion of total variance that is explained by the fixed effects only, whereas conditional *R* ^2^ represents the proportion of total variance that is explained by the fixed and the random effects (Schielzeth, 2013). A Cohen's *f* ^2^ type effect size was also calculated for each model (not available for multilevel Poisson models) by running the nested models for each predictor, and dropping the predictors one at a time. The corresponding changes in the marginal and conditional *R* ^2^ values from dropping each predictor were used to calculate the Cohen's *f* ^2^ type effect size:

$$\frac{R_{\mathrm{AB}}^{2}-R_{A}^{2}}{1-R_{\mathrm{AB}}^{2}}$$

where $R_{\mathrm{AB}}^{2}$ refers to the marginal *R* ^2^ from the full model, and $R_{A}^{2}$ is the marginal *R* ^2^ from the restricted model (i.e., after dropping the relevant predictor).

Between‐ and within‐person levels were tested simultaneously in all models. Between‐person levels examine differences between individuals, whereas within‐person levels examine whether daily deviations in TST/SE, relative to the individual's average TST/SE, predict next‐day outcomes. TST/SE variables were separated into two components: the between‐person component was calculated as the individual mean for each participant, and the daily, within‐person component as the difference between daily values and the individual mean. In order to provide a stronger test of directionality, all models controlled for the previous‐day, within‐person level outcome (Kalmbach, Pillai, Roth, & Drake, 2014). For example, sleep diary TST was tested as a predictor of next‐day morning KSS, controlling for previous‐day morning KSS. The fully adjusted models adjusted for the previous day's outcome, the aforementioned covariates, and school/ vacation status. School/vacation status was added as a moderator to the model. All sleep variables, as well as VAFS scores (both morning and afternoon) were winsorized (1%) for analyses to address a few extreme values.

## Results

### Descriptive statistics

A total of 205 adolescents (54.1% females, *M*_age_ *± SD* = 16.92 ± 0.87 years) completed the study, providing 4,868 (*M ± SD =* 24.4 ± 7.9 days/person) and 5,231 (*M ± SD =* 26.2 ± 5.4 days/person) nights of usable actigraphy and diary sleep data, respectively. Completion rates were high: 93.2% of morning surveys, 80.7% of afternoon surveys, 86.9% of actigraphy sleep episodes, and 79.0% of the PVT were available for analyses. While we did not query reasons of not completing some daily measures, missing data on actigraphy sleep were largely from faulty hardware. The number of daily observations ranged from 2,997 to 4,678 across models due to missing data and cross‐lags.

Sociodemographic variables are presented in Table 1. Most participants were Asian (55.6%), and were born in Australia (66.3%). A small proportion of participants self‐reported current depressive (4.39%), anxiety (5.85%), or sleep (11.2%) disorders. All analyses were rerun after excluding participants with current depressive and/or anxiety disorders, and/or sleep disorders. Results of the sensitivity analysis indicated minimal changes after excluding these participants (≤0.01 change to the coefficients and confidence intervals). These participants were therefore included in the final analyses to maintain generalizability of results to a community sample of adolescents.

Descriptive statistics for sleep and outcome variables are presented in Table 2. Participants' average actigraphy and diary TST over the study were 6.84 and 7.85 hr, respectively; average actigraphy and diary SE over the study were 81.9% and 92.6%, respectively. Compared to vacation, school term was associated with shorter TST (both actigraphy and diary; all *p* < .001), higher SE (actigraphy only; *p* < .001), and higher self‐reported sleepiness and fatigue (both morning and afternoon; all *p* < .001). During the vacation, adolescents' PVT performance was worse in most PVT variables, including a greater number of false starts and lapses (both *p* < .001), and slower reaction times (for all responses and the slowest 10% of responses; both *p* < .048).

**Table 2:** Descriptive statistics for sleep and outcome variables across school term and vacation

Study variables	*M* (*SD*)			*p* (Cohen's *d*)
	Entire study period	School term	Vacation	
Actigraphy sleep				
Total sleep time (hr)	6.84 (1.30)	6.50 (0.72)	7.02 (0.80)	**<.001 (0.78)**
Sleep efficiency (%)	81.9 (7.03)	82.23 (4.97)	81.59 (5.12)	**< .001 (0.25)**
Diary sleep				
Total sleep time (hr)	7.85 (1.50)	7.48 (0.78)	8.14 (0.84)	**<.001 (0.89)**
Sleep efficiency (%)	92.60 (8.55)	92.57 (4.95)	92.57 (5.80)	**.490 (0.05)**
Karolinska Sleepiness Scale				
Morning	4.17 (2.00)	4.49 (1.28)	3.86 (1.32)	**<.001 (0.64)**
Afternoon	3.87 (1.92)	4.06 (1.22)	3.70 (1.22)	**<.001 (0.39)**
Visual Analog of Fatigue Scale				
Morning	32.58 (24.16)	37.08 (16.23)	28.37 (16.49)	**<.001 (0.79)**
Afternoon	32.31 (24.98)	35.55 (16.73)	29.34 (17.46)	**<.001 (0.56)**
Psychomotor Vigilance Task				
Mean reciprocal reaction time (1/s)	4.48 (0.61)	4.43 (0.47)	4.42 (0.60)	.**034 (0.16)**
Mean reciprocal reaction time of slowest 10% responses (1/s)	2.92 (0.90)	2.85 (0.62)	2.83 (0.82)	.**048 (0.15)**
Mean reaction time of fastest 10% responses (s)	179.31 (17.09)	179.99 (15.80)	178.57 (18.42)	.466 (0.05)
Number of lapses (reaction times > 355 ms)	2.71 (3.93)	2.58 (3.58)	2.84 (4.25)	**<.001 (0.18)**
Number of false starts (reaction times < 100 ms)	3.36 (3.52)	3.16 (3.28)	3.56 (3.74)	**<.001 (0.08)**

ICC, Intraclass correlation coefficients. Karolinska Sleepiness Scale (Kaida et al., 2006) scores range from 1 to 10 (1 = e*xtremely alert and* 10 = e*xtremely sleepy, cannot stay awake*). Visual Analog of Fatigue Scale (Monk, 1989) scores range from 1 to 100 (higher scores indicating higher levels of fatigue). Total sleep time and sleep efficiency (actigraphy and diary), and Visual Analog of Fatigue scores (both morning and afternoon) were winsorized at 1%. The *p*‐values presented are based on paired samples *t*‐tests (Poisson regression models for number of lapses and false starts), between school term and vacation, conducted after averaging values for each participant across each 2‐week interval. Bolded values indicate significance at alpha‐level of .05.

School/vacation status was added as a moderator. Interactions terms were nonsignificant in all models and were subsequently dropped from all models. All subsequent main effect adjusted models included school/vacation status as covariate.

### Daily fluctuations in sleep duration and efficiency predicted better next‐day sustained attention

Table 3 shows the cross‐lagged multilevel models with actigraphy and diary TST and SE predicting PVT performance. See Table S1 for full results of the adjusted model including results for all covariates. On the between‐person level, higher self‐reported SE is associated with fewer false starts (*p* = .02). Specifically, each additional 1% higher self‐reported average SE was associated with a 2% decrease in the average number of false starts. No other significant between‐person associations were found between sleep and PVT variables.

**Table 3:** Multilevel models with sleep duration and efficiency predicting next‐day psychomotor vigilance task outcomes

	Mean 1/RT	Mean 1/RT (slowest 10%)	Mean RT (fastest 10%)	Lapses	False starts
Actigraphy TST (adjusted for covariates; *n* = 191, # of observations = 2,970)					
Between	0.02 [−0.09, 0.13], <0.01	0.07 [−0.08, 0.21], <0.01	−1.03 [−4.77, 2.71], <0.01	−0.05 [−0.23, 0.14] 0.96	0.09 [−0.06, 0.24] 1.09
Within (fixed)	0.02*** [0.01, 0.03], **<0.01**	0.03*** [0.01, 0.05], **<0.01**	−0.59** [−1.01, −0.16], **<0.01**	−0.03* [−0.05, 0.007] **0.97**	0.001 [−0.02, 0.02] 1.00
Actigraphy TST (not adjusted for covariates; *n* = 196, # of observations = 3,188)					
Between	0.02 [−0.09, 0.13], <0.01	0.11 [−0.04, 0.25], 0.01	−0.16 [−3.73, 3.40], <0.01	−0.06 [−0.22, 0.10]^§^ 0.90	0.04 [−0.06, 0.14]^§^ 1.15
Within (fixed)	0.03*** [0.02, 0.04], **<0.01**	0.04*** [0.02, 0.06], **<0.01**	−0.79*** [−1.21, −0.38], **<0.01**	−0.03 [−0.07, 0.004]^§^ 0.96	0.0002 [−0.03, 0.03]^§^ 0.99
Actigraphy SE (adjusted for covariates; *n* = 192, # of observations = 2,997)					
Between	0.01 [−0.003, 0.03], <0.01	0.02 [0.001, 0.04], 0.01	−0.33 [−0.83, 0.17], 0.01	−0.02 [−0.05, 0.001] 0.98	0.01 [−0.01, 0.03] 0.94
Within (fixed)	0.003* [0.001, 0.005], **<0.01**	0.002 [0.002, 0.01], <0.01	−0.15*** [−0.24, −0.07], **<0.01**	0.001 [−0.01, 0.004] 1.00	0.001[−0.004, 0.01] 0.98
Actigraphy SE (not adjusted for covariates; *n* = 196, # of observations = 3,188)					
Between	0.01 [−0.01, 0.02], <0.01	0.01 [−0.01, 0.03], 0.01	−0.05 [−0.55, 0.46], <0.01	−0.01 [−0.03, 0.01]^§^ 0.98	0.01 [−0.01, 0.02]^§^ 0.98
Within (fixed)	0.003* [0.001, 0.005], **<0.01**	0.002 [−0.002, 0.01], <0.01	−0.15*** [−0.24, −0.07], **<0.01**	−0.003 [−0.01, 0.01]^§^ 1.00	0.001 [−0.01, 0.01]^§^ 1.00
Diary TST (adjusted for covariates; *n* = 199, # of observations = 3,232)					
Between	−0.01 [−0.10, 0.08], 0.01	−0.02 [−0.15, 0.10], <0.01	0.12 [−3.00, 3.24], <0.01	0.03 [−0.12, 0.18] 1.04	−0.03 [−0.15, 0.09] 1.04
Within (fixed)	0.01** [0.01, 0.02], **<0.01**	0.03** [0.01, 0.04], **<0.01**	−0.28 [−0.63, 0.08], <0.01	−0.02 [−0.04, 0.004] 0.98	0.01 [−0.01, 0.03] 0.98
Diary TST (not adjusted for covariates; *n* = 203, # of observations = 3,422)					
Between	0.002 [−0.09, 0.09], <0.01	0.05 [−0.07, 0.18], <0.01	0.29 [−2.88, 3.46], <0.01	−0.01 [−0.16, 0.14]^†^ 0.94	−0.05 [−0.14, 0.04]^†^ 0.94
Within (fixed)	0.02*** [0.01, 0.03], **<0.01**	0.03*** [0.01, 0.04], **<0.01**	−0.46* [−0.81, −0.10], **<0.01**	−0.02 [−0.05, 0.01]^†^ 0.98	0.002 [−0.02, 0.03]^†^ 0.98
Diary SE (adjusted for covariates; *n* = 199, # of observations = 3,229)					
Between	0.01 [−0.01, 0.02], <0.01	0.01 [−0.01, 0.03], <0.01	−0.06 [−0.53, 0.42], <0.01	−0.01 [−0.04, 0.01] 0.99	−0.02** [−0.04, −0.01] **0.98**
Within (fixed)	0.001 [−0.001, 0.002], <0.01	0.001 [−0.002, 0.004], <0.01	−0.02 [−0.08, 0.04], <0.01	−0.001 [−0.001, 0.003] 1.00	0.004* [−0.0004, 0.01] 1.00
Diary SE (not adjusted for covariates; *n* = 203, # of observations = 3,419)					
Between	0.01 [−0.01, 0.02], 0.01	0.02 [−0.001, 0.04], 0.01	−0.14 [−0.62, 0.34], <0.01	−0.01 [−0.04, 0.01]^†^ 0.98	−0.02* [−0.03, −0.003] ^†^ 0.98
Within (fixed)	0.001 [−0.001, 0.003], <0.01	0.001 [−0.002, 0.004], <0.01	−0.03 [−0.09, 0.03], <0.01	−0.001 [−0.01, 0.004]^†^ 1.00	0.003 [−0.002, 0.01]^†^ 1.00

SE, sleep efficiency; TST, total sleep time. Lapses were defined as reaction times >355 ms, false starts were defined as reaction times <100 ms. Values presented are unstandardized coefficients (95% confidence intervals), Cohen's *f* ^2^ (not available for Poisson models). For multilevel Poisson models, the unstandardized coefficients are presented on the log scale. RT, reaction time. *p < .05, **p < .01, and ***p < .001. Bold indicates a significant within‐person or between‐person association. §represents *n* = 192/# of observations = 2,997 for this model. †represents *n* = 199/# of observations = 3,232 for this model. Random effects for within‐person level predictor were dropped from all models due to convergence issues and are therefore not presented; Cohen's *f* ^2^ are presented for the models with random effects for the within‐person predictor. The following covariates were included in all models: previous day/lagged outcome, school/vacation, body mass index, born in Australia, sex, year level, race, work status, parental marital status, study day, day of the week, chronotype, as well as self‐reported effort, motivation and distraction when completing the cognitive tasks.

Most significant associations were on the within‐person level, indicating that adolescents performed better on the PVT following nights with higher‐than‐average sleep duration and efficiency. Nights with longer‐than‐average TST (actigraphy and diary) predicted faster reaction times the following day (all responses *p* ≤ .001; slowest 10% of responses *p* ≤ .001; fastest 10% of responses *p* = .006). Taking the example of actigraphy TST predicting mean reaction time of the fastest 10% of responses, this indicates that after adjusting for 13 covariates and the lagged outcome, 1 hr longer‐than‐usual actigraphy TST significantly predicted 0.59‐s faster reaction time on the fastest 10% of responses.

Nights with higher‐than‐average actigraphy SE, also predicted faster mean reaction time (for all responses and for the fastest 10% of responses; both *p* < .001). No significant associations emerged for diary SE at the within‐person level.

### Daily fluctuations in sleep duration and efficiency predicted lower self‐reported sleepiness and fatigue the following morning and afternoon

Table 4 shows the cross‐lagged multilevel models with actigraphy and diary TST and SE predicting self‐reported sleepiness and fatigue in the mornings and afternoons. Refer to Table S2 for results of the adjusted model, including results of the covariates. On the between‐person level, higher diary SE was associated with lower self‐reported sleepiness in the mornings (*p* < .001). After adjusting for covariates, no other significant associations were found on the between‐person level.

**Table 4:** Multilevel models with sleep duration and efficiency predicting next‐day sleepiness and fatigue

	KSS		VAFS	
	Morning	Afternoon	Morning	Afternoon
Actigraphy TST (adjusted for covariates)				
Between	0.12 [−0.14, 0.38], <0.01	−0.01 [−0.30, 0.27], <0.01	1.00 [−2.27, 4.27], <0.01	−1.83 [−5.45, 1.79], <0.01
Within (fixed)	−0.14*** [−0.19, −0.10], **0.01**	−0.19*** [−0.24, −0.14], **0.01**	−2.55*** [−3.14, −1.95], **0.01**	−2.58*** [−3.19, −1.98], **0.01**
Within (random)	**–**	–	0.02**	–
*n*, # of observations	196, 4,096	194, 3,272	196, 4,130	194, 3,257
Actigraphy TST (not adjusted for covariates)				
Between	0.20 [−0.06, 0.45], <0.01	0.17 [−0.09, 0.43], <0.01	1.43 [−1.78, 4.64], <0.01	0.77 [−2.78, 4.33], <0.01
Within (fixed)	−0.13*** [−0.18, −0.09], **0.01**	−0.19*** [−0.24, −0.14], **0.01**	−2.52*** [−3.11, −1.93], **0.01**	−2.57*** [−3.17, −1.98], **0.01**
Within (random)	–	–	0.02*	–
*n*, # of observations	196, 4,096	194, 3,272	196, 4,130	194, 3,257
Actigraphy SE (adjusted for covariates)				
Between	−0.02 [−0.05, 0.02], <0.01	−0.02 [−0.06, 0.02], <0.01	0.08 [−0.37, 0.52], <0.01	0.12 [−0.37, 0.62], <0.01
Within (fixed)	−0.01* [−0.02, −0.001], **<0.01**	−0.004 [−0.01, 0.01], <0.01	−0.11* [−0.22, −0.01], **<0.01**	−0.12 [−0.25, 0.01], <0.01
Within (random)	–	–	–	<0.01
*n*, # of observations	196, 4,096	193, 3,244	196, 4,130	194, 3,257
Actigraphy SE (not adjusted for covariates)				
Between	0.001 [−0.04, 0.04], <0.01	−0.01 [−0.04, 0.03], <0.01	0.23 [−0.22, 0.69], <0.01	0.31 [−0.19, 0.80], <0.01
Within (fixed)	−0.01* [−0.02, −0.001], **<0.01**	0.01 [−0.01, 0.01], <0.01	−0.12* [−0.22, −0.02], **<0.01**	−0.13 [−0.26, −0.003], <0.01
Within (random)	–	–	–	<0.01
*n*, # of observations	196, 4,096	194, 3,272	196, 4,130	194, 3,257
Diary TST (adjusted for covariates)				
Between	−0.12 [−0.34, 0.10], <0.01	−0.15 [−0.37, 0.07], <0.01	−0.14 [−2.88, 2.60], <0.01	−0.94 [−3.91, 2.04], <0.01
Within (fixed)	−0.16*** [−0.19, −0.12], **0.01**	−0.19*** [−0.24, −0.15], **0.02**	−2.98*** [−3.48, −2.48], **0.03**	−2.51*** [−3.06, −1.96], **0.02**
Within (random)	–	–	0.02***	0.01
*n*, # of observations	204, 4,645	202, 3,527	204, 4,683	202, 3,523
Diary TST (not adjusted for covariates)				
Between	−0.17 [−0.39, 0.06], <0.01	−0.15 [−0.38, 0.08], <0.01	−1.28 [−4.11, 1.54], <0.01	−2.00 [−5.09, 1.10], <0.01
Within (fixed)	−0.15*** [−0.19, −0.12], **0.01**	−0.20*** [−0.24, −0.16], **0.02**	−2.96*** [−3.46, −2.47], **0.03**	−2.51*** [−3.06, −1.96], **0.02**
Within (random)	–	–	0.02***	0.01
*n*, # of observations	204, 4,645	202, 3,527	204, 4,683	202, 3,523
Diary SE (adjusted for covariates)				
Between	−0.07*** [−0.10, −0.03], **0.02**	−0.03 [−0.07, 0.001], 0.01	−0.40 [−0.83, 0.02], 0.01	0.04 [−0.42, 0.51], <0.01
Within (fixed)	−0.01*** [−0.02, −0.01], **<0.01**	−0.01** [−0.02, −0.004], **<0.01**	−0.25*** [−0.34, −0.15], **0.01**	−0.13** [−0.22, −0.04], **<0.01**
Within (random)	–	–	0.02***	–
*n*, # of observations	204, 4,640	201, 3,496	204, 4,678	202, 3,520
Diary SE (not adjusted for covariates)				
Between	−0.08*** [−0.11, −0.04], **0.04**	−0.05** [−0.09, −0.02], **0.02**	−0.58** [−1.00, −0.16], **0.01**	−0.37 [−0.83, 0.10], <0.01
Within (fixed)	−0.01*** [−0.02, −0.01], **<0.01**	−0.01** [−0.02, −0.01], **<0.01**	−0.25*** [−0.34, −0.16], **0.01**	−0.13** [−0.22, −0.04], **<0.01**
Within (random)	–	–	0.02***	–
*n*, # of observations	204, 4,640	202, 3,524	204, 4,678	202, 3,520

Values presented are unstandardized coefficients [95% confidence intervals], Cohen's *f* ^2^. SE, sleep efficiency; TST, total sleep time. Values in the *n, #* row are number of participants, number of observations. KSS, Karolinska Sleepiness Scale (Kaida et al., 2006) scores range from 1 to 10 (1 = extremely alert and 10 = extremely sleepy, cannot stay awake), VAFS, Visual Analog of Fatigue Scale (Monk, 1989) scores range from 1 to 100 (higher scores indicating higher levels of fatigue). *p < .05, **p < .01, and ***p < .001. Bold indicates a significant within‐person or between‐person association. Random effects for within‐person level predictor and lagged outcomes were included in all of the initial models, and subsequently dropped if the model did not converge (represented by –); Cohen's *f* ^2^ are presented for the models with random effects for the within‐person predictor. The following covariates were included in all models: previous day/lagged outcome, school/vacation, body mass index, born in Australia, sex, year level, race, work status, parental marital status, study day, day of the week, and chronotype.

At the within‐person level, longer TST (actigraphy and diary) predicted lower self‐reported sleepiness *and* fatigue the following day, both in the mornings and afternoons (all *p* < .001). Taking the example of diary TST with fatigue, an hour longer‐than‐average diary TST significantly predicted 2.98‐ and 2.51‐point lower fatigue (on a scale of 0–100) the following morning and afternoon, respectively.

The associations between SE with self‐reported sleepiness and fatigue differed according to the sleep measure (actigraphy vs. diary), and the time of day (morning ves. afternoon). Higher‐than‐average diary SE predicted lower self‐reported sleepiness and fatigue the following morning and afternoon (all *p* ≤ .005), whereas higher‐than‐average actigraphy SE predicted lower self‐reported sleepiness and fatigue, in the mornings only (all *p* ≤ .035).

Tables 3 and 4 present results from both the adjusted and the unadjusted models. In three models, the associations were only significant in the unadjusted model: Between‐persons, adolescents with higher overall diary SE also had lower afternoon self‐reported sleepiness and lower morning fatigue (all *p* ≤ .008). Within‐persons, nights with higher‐than‐average diary TST were associated with faster mean reaction times on the fastest 10% of responses (*p* = .012). These were no longer significant in the adjusted model, indicating that the sleep predictor did not make unique contributions beyond the lagged outcome and covariates. Refer to ‘Supporting Information’ for Tables S1 and S2 present results of the fully adjusted model, including results of each of the covariates and the lagged outcome.

## Discussion

This study investigated the associations between daily sleep and daytime alertness and fatigue over 28 days of relatively restricted and unrestricted sleep opportunities. Daily fluctuations in sleep duration and efficiency predicted adolescents' daytime attention, sleepiness, and fatigue on a day‐to‐day basis. Specifically, longer‐ and higher‐than usual sleep efficiency predicted lower self‐reported sleepiness and fatigue the following morning and afternoon and better sustained attention the following afternoon. Therefore, all hypotheses were supported, except for the hypothesis regarding the moderating effect of school term/vacation period, where these associations did not differ between school term or vacation periods.

### Daily fluctuations in sleep predict self‐report and objective alertness and fatigue

The within‐person findings between daily sleep and self‐report sleepiness and fatigue are consistent with the few daily studies conducted in this age group. For example, when adolescents retrospectively recalled their daily fatigue each evening, nights with shorter‐than‐usual self‐report sleep predicted higher next‐day fatigue (Fuligni & Hardway, 2006). Our findings extended these studies showing that adolescents who had shorter‐ and poorer‐than‐usual sleep (both estimated via self‐report and actigraphy) reported higher self‐reported sleepiness and fatigue levels that lasted beyond the mornings and extended into the afternoons. The lack of between‐person findings is consistent with previous research (Könen et al., 2015). This may be due to most late adolescents facing similar challenges such as peaking in delayed sleep timing, early school start times, studying and extracurricular activity later in the day (Carskadon, 2011; Wittmann, Dinich, Merrow, & Roenneberg, 2006).

Adolescents recorded better sustained attention following nights with longer‐ and higher‐than‐usual sleep efficiency. Our findings further support and extend the current literature, demonstrating that sacrificing sleep to study can have unintended negative consequences on daytime functional outcomes (e.g., Gillen‐O'Neel et al., 2013). Further, as these associations did not differ between school terms and vacations, these findings suggest that even during long periods of relatively unrestricted sleep opportunities, daily fluctuations in sleep continued to be relevant for adolescents' ability to sustain attention on a daily basis. It is worth noting that these effect sizes were relatively small, and there were few notable findings on lapses or false starts. It is likely that adolescents may not be aware of these small functional impact in everyday life. The potential differences in perceived versus actual sleep, as well as perceived and actual functional impact may also contribute to some differences in findings on self‐report and actigraphy‐derived sleep. For instance, actigraphy but not self‐report sleep efficiency predicted next‐day reaction speed.

### Limitations and directions for future research

These findings should be interpreted with consideration of the following limitations. Firstly, we examined nocturnal sleep in the study, and did not consider naps. Evidence from simulated studies suggest that afternoon naps may improve perceived sleepiness and reduce the number of lapses on the PVT (Lo et al., 2019). Although naps were not common in this sample (participants self‐reported naps in 0.1% of all sleep episodes) and were not included in sleep calculations, these findings may not generalize to adolescents and cultures who habitually nap in the afternoons (Paraskakis et al., 2008). Future studies are needed to ascertain the benefits of naps for adolescents' daytime alertness and fatigue on a day‐to‐day basis. Secondly, as this was a community sample of relatively healthy adolescents, only a small proportion of participants reported current sleep and/or mental health conditions and neurodevelopmental conditions were not directly assessed. Therefore, findings may not generalize to adolescents with such conditions. Thirdly, because of the unsupervised naturalistic conditions for the PVT, we cannot be certain if all trials were completed per the instructions (e.g., sat upright). Nevertheless, after completing the PVT, participants self‐reported effort, motivation, and distraction (1–10 scale) when completing the cognitive tasks for that day. These self‐ratings were adjusted in all analyses with PVT as the outcome. Since the order of school term and vacation period was not randomized (all school then vacation), this might have led to order effects in the results. Finally, as daytime functioning and alertness are impacted by circadian rhythms (Wright, Lowry, & LeBourgeois, 2012), and although we controlled for chronotype in this study, differential findings on morning versus afternoon measures may represent a time‐of‐day effect, which requires further investigation.

## Conclusion and implications

The current study demonstrates the importance of daily fluctuations in sleep for adolescents' alertness and fatigue. Daily sleep consistently predicted self‐report sleepiness and fatigue and objective measures of attention, highlighting the acute effects of nighttime sleep on next‐day functioning and alertness state. Further, these associations were observed after controlling for relevant covariates and alertness and fatigue the previous day, providing strong evidence that maintaining and improving adolescents' sleep duration and sleep efficiency on a daily basis could support alertness and reduce sleepiness and fatigue in this age group. Protecting adolescents' daily sleep may have potential real‐world implications for road safety. Adolescents had a 0.59‐s faster reaction time (on the fastest 10% of responses) following nights with 1 hr longer‐than‐usual sleep. In the context of road safety, a vehicle driving at 100 km/h travels an additional 16.4 m (approximately 2–4‐car‐lengths) in 0.59 s. A potential increased stopping distance of 2 to 4‐car‐lengths in emergency braking situations is particularly significant for this age group as adolescents are overrepresented in sleep‐related motor vehicle crashes (Danner & Phillips, 2008; Sarma, Carey, Kervick, & Bimpeh, 2013). Findings from this study add to considerable evidence highlighting the substantial benefits of increasing adolescents' sleep for road safety, including a 17% decrease in motor vehicle crash rates after school start times were delayed (Danner & Phillips, 2008).

Finally, not only should adolescent sleep interventions aim to improve adolescents' sleep duration and efficiency *overall*, prioritizing consistency, avoiding large deviations from typical sleep, and protecting adolescents' sleep on a *daily* basis should also be targeted. Specifically, these findings can serve as educational information to motivate adolescents to obtain longer and better sleep for measurable next‐day functioning benefits. Additionally, these results can serve as a caution against sacrificing sleep for other activities (e.g., study), which is particularly common in older adolescents (Adam, Snell, & Pendry, 2007).

## Ethical considerations

The project was approved by Monash University's Human Research Ethics Committee (project ID: 10005). Informed consent was received from parents or guardians for all participants, and all participants provided informed assent to participate in the project.

**Key points**

- Current understanding of *day‐to‐day* associations between adolescents' sleep and self‐report sleepiness and fatigue and objective alertness and attention is limited, particularly under naturalistic sleep restricted and unrestricted sleep opportunities (i.e., school term and vacations, respectively).
- In a community sample of older adolescents, shorter and poorer than usual sleep predicted poorer sustained attention, and higher self‐reported sleepiness and fatigue the next day (i.e., within‐person associations).
- Study findings highlight the short‐term effects of daily sleep on adolescents' next‐day alertness and fatigue. Given that most adolescents experience chronic sleep restriction, and increasing sleep on school days is difficult given early school start times, interventions and educational efforts can aim to protect day‐to‐day sleep to benefit adolescents' alertness and fatigue.
- The magnitude of the associations suggests real‐world implications on road safety and classroom learning outcomes.

## Supporting information


**Table S1.** Covariates included in the multilevel models with sleep predicting psychomotor vigilance task outcomes.
**Table S2.** Covariates included in the multilevel models with sleep predicting sleepiness and fatigue.

## Data Availability

Data may be available upon reasonable request made to the corresponding author.
