# Supplementary material for: Daily fluctuations in adolescents' sleep predict next‐day attention, sleepiness, and fatigue: an ecological momentary assessment study over 28 days
Source: J Child Psychol Psychiatry. 2024 Dec 1;66(5):686–96. doi: 10.1111/jcpp.14076 (PMC12018295; doi:10.1111/jcpp.14076)
Supplement: Supplementary file 1 — Table S1. Covariates included in the multilevel models with sleep predicting psychomotor vigilance task outcomes. Table S2. Covariates included in the multilevel models with sleep predicting sleepiness and fatigue. [file JCPP-66-686-s001.docx]

**Table S1**

**Table S1.** Covariates Included in the Multilevel Models with Sleep Predicting Psychomotor Vigilance Task Outcomes

|  | **Mean 1/RT** | **Mean 1/RT**  **(slowest 10%)** | **Mean RT**  **(fastest 10%)** | **Lapses** | **False Starts** |
| --- | --- | --- | --- | --- | --- |
| **Actigraphy TST** | | | | | |
| Within Lagged Outcome (fixed) | **0.20*** [0.16, 0.24],**  **0.01** | **0.10*** [0.05, 0.14],**  **<.001** | **0.14*** [0.09, 0.18]**,  **<.001** | **0.07*** [0.03, 0.11]** | **0.09*** [ 0.05, 0.14]** |
| Within Lagged Outcome (random) | **0.03***** | **0.03***** | **0.01**** | – | -- |
| School Term | -0.0003 [-0.04, 0.04],  <.001 | -0.01 [-0.09, 0.07],  <.001 | 0.33 [-1.46, 2.12],  <.001 | 0.03 [-0.08, 0.13] | 0.05 [-0.05, 0.15] |
| Body Mass Index | 0.01 [-0.01, 0.02],  <.001 | 0.01 [-0.01, 0.03],  <.001 | -0.26 [-0.79, 0.27],  <.001 | -0.01 [-0.04, 0.02] | **0.03** [ 0.01, 0.05]** |
| Born in Australia | -0.01 [-0.16, 0.13],  <.001 | -0.09 [-0.29, 0.11],  <.001 | -0.95 [-6.00, 4.09],  <.001 | 0.19 [-0.06, 0.43] | -0.06 [-0.26, 0.14] |
| Female | **-0.20** [-0.34, -0.06],**  **0.04** | -0.15 [-0.34, 0.04],  0.01 | **6.95** [2.13, 11.78],**  **0.03** | 0.19 [-0.05, 0.42] | -0.02 [-0.21, 0.18] |
| Year Level | 0.08 [-0.01, 0.17],  0.01 | -0.02 [-0.14, 0.11],  <.001 | **-4.11* [-7.22, -1.00],**  **0.03** | 0.03 [-0.12, 0.18] | -0.11 [-0.23, 0.02] |
| Non-white | 0.09 [-0.066, 0.24],  0.01 | 0.00 [-0.21, 0.20],  <.001 | -4.85 [-10.03, 0.34],  0.01 | 0.07 [-0.18, 0.33] | -0.05 [-0.26, 0.16] |
| Working | -0.06 [-0.14, 0.02],  0.01 | -0.08 [-0.19, 0.03],  0.01 | 1.12 [-1.62, 3.87],  0.01 | 0.09 [-0.05, 0.22] | 0.01 [-0.10, 0.12] |
| Parental Marital Status | 0.003 [-0.17, 0.17], <.001 | -0.01 [-0.24, 0.22],  <.001 | -0.94 [-6.84, 4.96],  <.001 | 0.03 [-0.26, 0.32] | -0.02 [-0.25, 0.22] |
| Study Day | -0.001 [-0.004, 0.001],  <.001 | -0.003 [-0.01, 0.002],  <.001 | -0.06 [-0.17, 0.05],  <.001 | **0.01* [0.00, 0.01]** | **0.01** [ 0.003, 0.02]** |
| Day of the Week  (effect size) | <.001 | <.001 | <.001 | – | – |
| Monday | 0.02 [-0.02, 0.06] | **0.08* [ 0.002, 0.1]** | -0.03 [-1.68, 1.63] | -0.09 [-0.18, 0.01] | 0.05 [-0.05, 0.15] |
| Tuesday | 0.03 [-0.01, 0.07] | 0.07 [-0.004, 0.14] | -1.48 [ -3.11, 0.15] | -0.09 [-0.18, 0.01] | 0.07 [-0.02, 0.16] |
| Wednesday | 0.01 [-0.03, 0.05] | 0.05 [-0.02, 0.12] | 0.01 [ -1.63, 1.65] | -0.06 [-0.16, 0.03] | 0.05 [-0.05, 0.14] |
| Thursday | **0.05* [0.01, 0.09]** | **0.08* [ 0.01, 0.15]** | -1.23 [-2.87, 0.42] | **-0.11* [-0.21, -0.01]** | 0.05 [-0.05, 0.14] |
| Saturday | 0.02 [-0.02, 0.06] | 0.01 [-0.07, 0.08] | -0.82 [-2.55, 0.90] | -0.02 [-0.12, 0.08] | 0.08 [-0.02, 0.18] |
| Sunday | **0.05* [0.01, 0.09]** | 0.07 [-0.005, 0.15] | **-1.84* [-3.55, -0.13]** | **-0.12* [-0.22, -0.02]** | **0.10* [ 0.003, 0.20]** |
| Effort | **0.05*** [0.04, 0.06],**  **0.03** | **0.07*** [0.06, 0.09],**  **0.02** | **-1.35*** [-1.76, -0.95],**  **0.01** | **-0.07***[-0.09, -0.05]** | **-0.03* [-0.05, -0.003]** |
| Motivation | 0.01 [-0.002, 0.02],  <.001 | 0.01 [-0.01, 0.02],  <.001 | -0.19 [-0.55, 0.18],  <.001 | -0.01 [-0.03, 0.01] | -0.01 [-0.03, 0.01] |
| Distraction | **-0.04*** [-0.04, -0.03],**  **0.02** | **-0.08*** [-0.09, -0.06],**  **0.04** | **0.70*** [0.46, 0.93],**  **0.01** | **0.10*** [ 0.08, 0.11]** | 0.003 [-0.01, 0.02] |
| Morningness Eveningness | **0.01* [ 0.001, 0.02],**  **0.02** |  | -0.27 [ -0.57, 0.03]  0.01 | -0.01 [-0.03, 0.003] | 0.002 [-0.01, 0.01] |
| *n,* # of observations | 191, 2970 | 192, 2997 | 192, 2997 | 191, 2976 | 192, 2997 |
| **Actigraphy SE** | | | | | |
| Within Lagged Outcome (fixed) | **0.21*** [0.17, 0.24],**  **0.02** | **0.09*** [0.05, 0.13],**  **<.001** | **0.14*** [0.09, 0.18],**  **<.001** | **0.07*** [ 0.03, 0.11]** | **0.09*** [ 0.05, 0.13]** |
| Within Lagged Outcome (random) | **--** | **<.001***** | **0.01**** | **--** | -- |
| School Term | -0.02 [-0.06, 0.03],  <.001 | -0.03 [-0.11, 0.05],  <.001 | 0.84 [ -0.92, 2.60],  <.001 | 0.05 [-0.05, 0.15] | 0.05 [-0.05, 0.16] |
| Body Mass Index | 0.005 [-0.01, 0.02],  <.001 | 0.01 [-0.01, 0.03],  <.001 | -0.23 [-0.75, 0.29],  <.001 | -0.01 [-0.03, 0.02] | **0.03** [ 0.01, 0.05]** |
| Born in Australia | 0.01 [-0.14, 0.16],  <.001 | -0.06 [-0.26, 0.14],  <.001 | -1.52 [-6.62, 3.59],  <.001 | 0.15 [-0.10, 0.40] | -0.08 [-0.28, 0.12] |
| Female | **-0.22** [-0.36, -0.09],**  **0.05** | -0.16 [-0.35, 0.03],  0.01 | **7.27** [2.55, 11.99],**  **0.04** | 0.21 [-0.02, 0.44] | 0.01 [-0.18, 0.20] |
| Year Level | **0.10* [ 0.004, 0.19],**  **0.02** | -0.01 [-0.13, 0.12],  <.001 | **-4.35** [-7.47, -1.23],**  **0.03** | 0.01 [-0.14, 0.17] | -0.08 [-0.21, 0.04] |
| Non-white | 0.07 [-0.08, 0.22],  <.001 | -0.02 [-0.22, 0.18],  <.001 | -4.60 [-9.69, 0.48],  0.01 | 0.08 [-0.16, 0.33] | -0.10 [-0.30, 0.10] |
| Working | -0.06 [-0.14, 0.02],  0.01 | -0.09 [-0.19, 0.02],  0.01 | 1.25 [-1.47, 3.98],  0.01 | 0.09 [-0.04, 0.23] | 0.01 [-0.10, 0.12] |
| Parental Marital Status | -0.03 [-0.20, 0.14],  <.001 | -0.02 [-0.26, 0.21],  <.001 | -0.59 [-6.48, 5.29],  <.001 | 0.05 [-0.24, 0.33] | -0.08 [-0.31, 0.16] |
| Study Day | -0.001 [-0.003, 0.002],  <.001 | -0.003 [-0.01, 0.002],  <.001 | -0.05 [-0.16, 0.06],  <.001 | **0.01* [ 0.001, 0.01]** | **0.01** [ 0.004, 0.02]** |
| Day of the Week  (effect size) | <.001 | <.001 | <.001 | – | – |
| Monday | 0.02 [-0.02, 0.06] | **0.08* [ 0.003, 0.15]** | -0.16 [-1.82, 1.50] | -0.09 [-0.19, 0.01] | 0.05 [-0.04, 0.15] |
| Tuesday | 0.03 [-0.01, 0.07] | 0.07 [-0.001, 0.14] | -1.52 [-3.14, 0.11] | -0.09 [-0.19, 0.004] | 0.07 [-0.03, 0.16] |
| Wednesday | 0.01 [-0.03, 0.05] | 0.05 [-0.02, 0.12] | -0.01 [-1.66, 1.63] | -0.07 [-0.16, 0.03] | 0.04 [-0.05, 0.14] |
| Thursday | 0.05* [0.01, 0.09] | 0.09* [ 0.01, 0.16] | -1.37 [-3.01, 0.27] | **-0.12* [-0.21, -0.02]** | 0.05 [-0.05, 0.14] |
| Saturday | 0.02 [-0.02, 0.06] | 0.02 [-0.05, 0.10] | -1.01 [-2.72, 0.70] | -0.03 [-0.13, 0.07] | 0.08 [-0.02, 0.18] |
| Sunday | 0.05** [0.01, 0.10] | 0.01 [-0.06, 0.08] | **-2.07* [-3.77, -0.37]** | **-0.13** [-0.23, -0.04]** | 0.10* [0.002, 0.20] |
| Effort | **0.05*** [0.04, 0.06],**  **0.03** | **0.07*** [0.06, 0.09],**  **0.02** | **-1.35*** [-1.76, -0.94],**  **0.01** | **-0.07***[-0.09, -0.05]** | -0.02 [-0.05, 0.001] |
| Motivation | 0.01 [-0.002, 0.02],  <.001 | 0.01 [-0.01, 0.02],  <.001 | -0.19 [-0.55, 0.18],  <.001 | -0.01 [-0.03, 0.01] | -0.01 [-0.03, 0.01] |
| Distraction | **-0.04*** [-0.04, -0.03],**  **0.02** | **-0.08*** [-0.09, -0.07],**  **0.04** | **0.71*** [0.47, 0.95],**  **0.01** | **0.10*** [ 0.08, 0.11]** | 0.003 [-0.01, 0.02] |
| Morningness Eveningness | **0.01* [ 0.001, 0.02],**  **0.02** | **0.01* [ 0.0001, 0.02]** | -0.29 [-0.59, 0.01]  0.02 | -0.01 [-0.03, 0.001] | 0.004 [-0.01, 0.02] |
| *n,* # of observations | 192, 2997 | 192, 2997 | 192, 2997 | 191, 2976 | 191, 2969 |
| **Diary TST** | | | | | |
| Within Lagged Outcome (fixed) | **0.21*** [0.16, 0.25],**  **<.001** | **0.09*** [0.05, 0.13],**  **<.001** | **0.15*** [0.11, 0.18],**  **0.01** | **0.06** [0.02, 0.10]** | **0.11*** [ 0.07, 0.16]** |
| Within Lagged Outcome (random) | **0.02***** | **0.02***** | -- | **--** | -- |
| School Term | -0.001 [-0.04, 0.04],  <.001 | -0.001 [-0.08, 0.08],  <.001 | 0.32 [-1.42, 2.07],  <.001 | 0.03 [-0.07, 0.13] | 0.06 [-0.04, 0.15] |
| Body Mass Index | 0.01 [-0.0067, 0.0231],  <.001 | 0.01 [-0.01, 0.03],  <.001 | -0.28 [-0.80, 0.24],  <.001 | -0.01 [-0.04, 0.01] | **0.03** [ 0.01, 0.05]** |
| Born in Australia | 0.02 [-0.12, 0.17],  <.001 | -0.08 [-0.28, 0.11],  <.001 | -1.46 [-6.42, 3.50],  <.001 | 0.17 [-0.07, 0.41] | -0.05 [-0.24, 0.15] |
| Female | **-0.19** [-0.33, -0.06],**  **0.04** | -0.16 [-0.34, 0.02], 0.02 | **7.57** [3.02, 12.12],**  **0.04** | 0.19 [-0.03, 0.41] | 0.05 [-0.13, 0.23] |
| Year Level | 0.08 [-0.01, 0.17],  0.01 | -0.02 [-0.14, 0.11],  <.001 | **-3.79* [-6.86, -0.72],**  **0.02** | 0.03 [-0.12, 0.18] | **-0.13* [-0.25, -0.01]** |
| Non-white | 0.05 [-0.09, 0.20],  <.001 | -0.05 [-0.25, 0.15],  <.001 | -4.69 [-9.74, 0.36],  0.01 | 0.11 [-0.13, 0.36] | -0.09 [-0.29, 0.10] |
| Working | -0.05 [-0.13, 0.03],  0.01 | -0.07 [-0.17, 0.04],  0.01 | 1.09 [-1.63, 3.81],  0.01 | 0.08 [-0.05, 0.21] | -0.003 [-0.11, 0.10] |
| Parental Marital Status | -0.05 [-0.22, 0.1191],  <.001 | -0.07 [-0.30, 0.16],  <.001 | -0.30 [-6.11, 5.51],  <.001 | 0.10 [-0.18, 0.38] | -0.02 [-0.25, 0.21] |
| Study Day | -0.001 [-0.003, 0.002],  <.001 | -0.002 [-0.01, 0.003],  <.001 | -0.09 [-0.20, 0.02],  <.001 | **0.01* [ 0.0002, 0.01]** | **0.01*** [ 0.005, 0.02]** |
| Day of the Week  (effect size) | <.001 | <.001 | <.001 | – | – |
| Monday | 0.02 [-0.02, 0.06] | **0.09* [ 0.02, 0.16]** | 0.71 [-0.91, 2.33] | **-0.11* [-0.21, -0.02]** | 0.03 [-0.06, 0.12] |
| Tuesday | 0.03 [-0.004, 0.07] | **0.08* [ 0.01, 0.15]** | -0.92 [-2.51, 0.68] | **-0.14** [-0.23, -0.04]** | 0.03 [-0.06, 0.12] |
| Wednesday | 0.01 [-0.03, 0.05] | 0.03 [-0.04, 0.10] | 0.50 [-1.11, 2.10] | -0.07 [-0.17, 0.02] | 0.03 [-0.06, 0.12] |
| Thursday | **0.05** [ 0.02, 0.09]** | **0.09* [ 0.02, 0.16]** | -0.81 [-2.43, 0.80] | **-0.13** [-0.23, -0.04]** | 0.01 [-0.08, 0.10] |
| Saturday | 0.014 [-0.03, 0.05] | 0.01 [-0.06, 0.08] | -0.37 [-2.03, 1.29] | -0.04 [-0.13, 0.06] | 0.07 [-0.02, 0.17] |
| Sunday | 0.04 [-0.001, 0.08] | 0.06 [-0.02, 0.13] | -0.99 [-2.64, 0.66] | **-0.12* [-0.22, -0.02]** | 0.08 [-0.02, 0.17] |
| Effort | **0.06*** [0.05, 0.07],**  **0.03** | **0.08*** [0.06, 0.10],**  **0.02** | **-1.48*** [-1.87, -1.09],**  **0.02** | **-0.08*** [-0.10, -0.05]** | **-0.02* [-0.04, -0.001]** |
| Motivation | **0.01* [0.0006, 0.02],**  **<.001** | 0.01 [-0.004, 0.03],  <.001 | -0.20 [-0.55, 0.15],  <.001 | -0.01 [-0.03, 0.01] | -0.01 [-0.03, 0.01] |
| Distraction | **-0.04*** [-0.04, -0.03],**  **0.03** | **-0.08*** [-0.09, -0.07],**  **0.04** | **0.72*** [0.49, 0.96],**  **0.01** | **0.09*** [ 0.08, 0.11]** | 0.004 [-0.01, 0.02] |
| Morningness Eveningness |  | **0.01* [ 0.001, 0.02]**  **0.02** | **-0.27 [-0.56, 0.02]**  **0.01** | -0.01 [-0.03, 0.002] | 0.005 [-0.01, 0.02] |
| *n,* # of observations | 199, 3232 | 199, 3232 | 199, 3232 | 198, 3204 | 199, 3232 |
| **Diary SE** | | | | | |
| Within Lagged Outcome (fixed) | **0.21*** [0.16, 0.25],**  **<.001** | **0.09*** [0.05, 0.13]**,  **<.001** | **0.15*** [0.11, 0.18],**  **0.01** | **0.06** [ 0.02, 0.10]** | **0.1*** [ 0.07, 0.16]** |
| Within Lagged Outcome (random) | **0.02***** | **0.02***** | -- | **--** | -- |
| School Term | -0.01 [-0.05, 0.03],  <.001 | -0.02 [-0.10, 0.05],  <.001 | 0.54 [-1.18, 2.26],  <.001 | 0.05 [-0.05, 0.15] | 0.05 [-0.05, 0.15] |
| Body Mass Index | 0.01 [-0.01, 0.02],  <.001 | -0.02 [-0.10, 0.05],  <.001 | -0.29 [-0.81, 0.23],  <.001 | -0.02 [-0.04, 0.01] | **0.03** [ 0.01, 0.05]** |
| Born in Australia | 0.02 [-0.12, 0.16],  <.001 | -0.08 [-0.27, 0.12],  <.001 | -1.54 [-6.52, 3.44],  <.001 | 0.16 [-0.09, 0.40] | -0.07 [-0.27, 0.12] |
| Female | -**0.19** [-0.32, -0.06],**  **0.04** | -0.15 [-0.33, 0.03],  0.01 | **7.55** [ 2.95, 12.15],**  **0.04** | 0.17 [-0.05, 0.40] | 0.01 [-0.17, 0.19] |
| Year Level | 0.09 [-0.001, 0.18],  0.01 | -0.004 [-0.13, 0.12],  <.001 | **-3.91* [-6.99, -0.82],**  **0.02** | 0.01 [-0.14, 0.16] | **-0.15* [-0.27, -0.03]** |
| Non-white | 0.07 [-0.08, 0.21],  <.001 | -0.06 [-0.26, 0.14],  <.001 | -4.64 [-9.72, 0.44],  0.01 | 0.13 [-0.12, 0.38] | -0.06 [-0.26, 0.13] |
| Working | -0.04 [-0.12, 0.04],  0.01 | -0.06 [-0.16, 0.05],  0.01 | 1.04 [-1.65, 3.74],  <.001 | 0.07 [-0.06, 0.20] | -0.01 [-0.11, 0.10] |
| Parental Marital Status | -0.02 [-0.19, 0.14], <.001 | -0.06 [-0.29, 0.7], <.001 | -0.45 [-6.26, 5.37],  <.001 | 0.09 [-0.19, 0.37] | -0.03 [-0.26, 0.19] |
| Study Day | -0.001 [-0.003, 0.002],  <.001 | -0.002 [-0.01, 0.003],  <.001 | -0.08 [-0.19, 0.02],  <.001 | **0.01* [0.001, 0.01]** | **0.01*** [0.004, 0.02]** |
| Day of the week  (effect size) | <.001 | <.001 | <.001 | – | – |
| Monday | 0.02 [-0.02, 0.06] | **0.09* [ 0.01, 0.16]** | 0.73 [-0.89, 2.35] | -0.11* [-0.21, -0.02] | 0.03 [-0.06, 0.13] |
| Tuesday | 0.03 [-0.004, 0.07] | **0.09* [ 0.02, 0.16]** | -0.93 [-2.52, 0.67] | **-0.14** [-0.23, -0.05]** | 0.03 [-0.06, 0.12] |
| Wednesday | 0.01 [-0.03, 0.05] | 0.03 [-0.04, 0.11] | 0.49 [-1.12, 2.10] | -0.07 [-0.17, 0.02] | 0.03 [-0.06, 0.12] |
| Thursday | **0.06** [ 0.02, 0.094]** | **0.09** [ 0.02, 0.17]** | -0.84 [-2.46, 0.77] | **-0.14** [-0.23, -0.04]** | 0.01 [-0.08, 0.10] |
| Saturday | 0.02 [-0.02, 0.06] | 0.02 [-0.05, 0.09] | -0.49 [-2.15, 1.17] | -0.05 [-0.14, 0.05] | 0.08 [-0.02, 0.17] |
| Sunday | **0.04* [ 0.004, 0.08]** | 0.06 [-0.01, 0.14] | -1.15 [-2.80, 0.50] | **-0.13** [-0.23, -0.04]** | 0.08 [-0.01, 0.18] |
| Effort | **0.06*** [0.05, 0.07],**  **0.03** | **0.08*** [0.06, 0.10],**  **0.02** | **-1.47*** [-1.86, -1.08],**  **0.02** | **-0.08*** [-0.10, -0.06]** | **-0.02* [-0.05, -0.003]** |
| Motivation | **0.01* [0.001, 0.02],**  **<.001** | 0.01 [-0.01, 0.03],  <.001 | -0.19 [-0.54, 0.16],  <.001 | -0.01 [-0.03, 0.01] | -0.01 [-0.03, 0.01] |
| Distraction | **-0.04*** [-0.04, -0.03],**  **0.03** | **-0.08*** [-0.09, -0.07],**  **0.04** | **0.73*** [0.49, 0.96],**  **0.01** | **0.09*** [ 0.08, 0.11]** | 0.004 [-0.01, 0.02] |
| Morningness Eveningness | **0.01* [ 0.001, 0.02]**  **0.02** | **0.01* [ 0.001, 0.01],**  **0.02** | **-0.27 [-0.56, 0.02]**  **0.01** | **-0.01 [-0.03, 0.001]** | 0.004 [-0.01, 0.01] |
| *n,* # of observations | 199, 3229 | 199, 3229 | 199, 3232 | 198, 3201 | 199, 3232 |
| *Note*. Values presented are unstandardised coefficients [95% confidence intervals], Cohen’s *f*^2^ (not available for Poisson models). For multilevel Poisson models, the unstandardized coefficients are presented on the log scale. RT = reaction time. **p < .*05*, **p < .*01, ****p* < .001. Bold indicates a significant association. Random effects for within-person level lagged outcomes were included in all the initial models, and subsequently dropped if the model did not converge (represented by --); Cohen’s *f*^2^ are presented for the models with random effects for the within-person predictor. For day of the week, a single Cohen’s *f*^2^ effect size is presented for the covariate before individual unstandardised coefficients and [95% confidence intervals] for each day; Friday was used as the reference level. | | | | | |

**Table S2**

**Table S2.** Covariates Included in the Multilevel Models with Sleep Predicting Sleepiness and Fatigue

|  | **KSS** | | **VAFS** | |
| --- | --- | --- | --- | --- |
|  | **Morning** | **Afternoon** | **Morning** | **Afternoon** |
| **Actigraphy TST** | | | | |
| Within Lagged Outcome (fixed) | **0.18*** [0.15, 0.21],**  **0.02** | **0.12*** [0.06, 0.16],**  **0.01** | **0.21*** [0.18, 0.24],**  **0.01** | **0.16*** [0.12, 0.19],**  **0.02** |
| Within Lagged Outcome (random) | **0.02***** | **0.02***** | **--** | -- |
| School Term | 0.07 [-0.12, 0.25],  <0.01 | -0.13 [-0.34, 0.08],  <0.01 | **2.64* [0.49, 4.79],**  **<0.01** | 1.29 [ -1.31, 3.89], <0.01 |
| Body Mass Index | 0.02 [-0.02, 0.06],  <0.01 | 0.02 [-0.02, 0.06],  <0.01 | -0.18 [-0.66, 0.30],  <0.01 | 0.24 [-0.28, 0.76],  <0.01 |
| Born in Australia | 0.14 [-0.23, 0.50],  <0.01 | 0.10 [-0.27, 0.47],  <0.01 | -1.06 [-5.62, 3.50],  <0.01 | -2.60 [ -7.45, 2.26],  <0.01 |
| Female | 0.31 [-0.03, 0.65],  0.01 | **0.43* [ 0.08, 0.79],**  **0.01** | **5.62* [1.32, 9.93],**  **0.01** | **7.74** [3.10, 12.38],**  **0.02** |
| Year Level | -0.09 [-0.31, 0.14],  <0.01 | 0.05 [-0.18, 0.28],  <0.01 | 0.39 [-2.37, 3.16],  <0.01 | **3.05* [0.08, 6.02], <0.01** |
| Non-white | **-0.39* [-0.76, -0.01],**  **0.01** | -0.34 [-0.72, 0.04],  0.01 | -4.62 [-9.31, 0.06],  0.01 | -4.71 [ -9.72, 0.30], 0.01 |
| Working | 0.06 [-0.14, 0.26],  <0.01 | 0.09 [-0.12, 0.29],  <0.01 | **3.61** [1.15, 6.07],**  **0.02** | **3.21* [0.54, 5.87],**  **0.01** |
| Parental Marital Status | 0.19 [-0.23, 0.62],  <0.01 | -0.08 [-0.52, 0.35],  <0.01 | 1.80 [-3.54, 7.13],  <0.01 | 0.75 [ -4.98, 6.49],  <0.01 |
| Morningness Eveningness | **-0.04*** [-0.07, -0.02],**  **0.03** | 0.003 [-0.02, 0.03],  <0.01 | **-0.48*** [-0.75, -0.20],**  **0.03** | 0.06 [ -0.23, 0.36],  <0.01 |
| Study Day | **-0.02** [-0.03, -0.01],**  **<0.01** | **-0.02* [-0.03, -0.003],**  **<0.01** | **-0.20**[-0.32, -0.07],**  **<0.01** | -0.11 [ -0.27, 0.05], <0.01 |
| Day of the week  (effect size) | <.001 | <.001 | <.001 | <.001 |
| Monday | **0.22* [0.05, 0.39]** | 0.039 [-0.16, 0.24] | **3.10** [1.14, 5.05]** | 1.74 [ -0.66, 4.13] |
| Tuesday | 0.10 [-0.08, 0.28] | -0.03 [-0.23, 0.16] | 0.81 [-1.21, 2.84] | 0.53 [ -1.85, 2.91] |
| Wednesday | 0.09 [-0.09, 0.27] | 0.04 [-0.16, 0.24] | 1.42 [-0.60, 3.45] | 1.60 [ -0.78, 3.98] |
| Thursday | 0.04 [-0.13, 0.22] | 0.003 [-0.19, 0.20] | 1.73 [-0.31, 3.77] | 0.39 [ -2.00, 2.78] |
| Saturday | 0.14 [-0.04, 0.32] | -0.07 [-0.27, 0.13] | 1.50 [-0.60, 3.61] | 0.15 [ -2.34, 2.64] |
| Sunday | 0.12 [-0.06, 0.30] | -0.06 [-0.27, 0.14] | 0.33 [-1.76, 2.41] | 1.04 [ -1.42, 3.50] |
| *n,* # of observations | 194, 4052 | 193, 3244 | 194, 4086 | 194, 3257 |
| **Actigraphy SE** | | | | |
| Within Lagged Outcome (fixed) | **0.18*** [0.14, 0.22],**  **0.02** | **0.11*** [0.07, 0.15],**  **0.01** | **0.20*** [0.17, 0.23],**  **0.03** | **0.15*** [0.11, 0.18],**  **0.01** |
| Within Lagged Outcome (random) | **0.02***** | **0.02***** | -- | -- |
| School Term | 0.18 [0.00, 0.37],  <0.01 | 0.01 [-0.20, 0.22],  <0.01 | **4.76*** [2.62, 6.91],**  **<0.01** | **3.31* [0.73, 5.89],**  **<0.01** |
| Body Mass Index | 0.02 [-0.01, 0.06],  <0.01 | 0.03 [-0.01, 0.07],  <0.01 | -0.13 [-0.60, 0.35],  <0.01 | 0.16 [-0.36, 0.68],  <0.01 |
| Born in Australia | 0.06 [-0.32, 0.43],  <0.01 | 0.06 [-0.32, 0.44],  <0.01 | -1.82 [-6.46, 2.81],  <0.01 | -2.26 [-7.25, 2.72],<0.01 |
| Female | **0.39* [ 0.05, 0.73],**  **0.01** | **0.46* [ 0.11, 0.82],**  **0.01** | **6.05** [1.81, 10.30],**  **0.02** | **6.83** [2.22, 11.44],**  **0.02** |
| Year Level | -0.10 [-0.32, 0.13],  <0.01 | 0.03 [-0.20, 0.26],  <0.01 | 0.52 [-2.28, 3.32],  <0.01 | **3.38* [0.36, 6.40],**  **0.01** |
| Non-white | **-0.41* [-0.78, -0.04],**  **0.01** | -0.35 [-0.73, 0.03],  0.01 | **-4.75* [-9.36, -0.14],**  **0.01** | -4.33 [-9.30, 0.65],  0.01 |
| Working | 0.05 [-0.15, 0.24],  <0.01 | 0.09 [-0.11, 0.29],  <0.01 | **3.41** [0.95, 5.88],**  **0.02** | **3.22* [0.54, 5.90],**  **0.01** |
| Parental Marital Status | 0.26 [-0.16, 0.69],  <0.01 | -0.08 [-0.52, 0.36],  <0.01 | 1.96 [-3.33, 7.24],  <0.01 | 0.49 [-5.30, 6.28],  <0.01 |
| Morningness Eveningness | **-0.04*** [-0.06, -0.02],**  **0.03** | 0.002 [-0.02, 0.02]  <0.01 | **-0.46*** [ -0.73, -0.19]**  **0.02** | 0.04 [ -0.26, 0.33], <0.01 |
| Study Day | **-0.02** [-0.03, -0.001],**  **<0.01** | **-0.01* [-0.03, -0.00001],**  **<0.01** | **-0.15* [-0.27, -0.02],**  **<0.01** | -0.08 [-0.24, 0.09],  <0.01 |
| Day of the Week  (effect size) | <.001 | <.001 | <.001 | <.001 |
| Monday | 0.23** [0.06, 0.40] | 0.04 [-0.16, 0.24] | **3.51*** [1.52, 5.49]** | 1.68 [-0.74, 4.10] |
| Tuesday | 0.09 [-0.08, 0.27] | -0.05 [-0.25, 0.15] | 0.90 [-1.15, 2.96] | 0.22 [-2.18, 2.63] |
| Wednesday | 0.07 [-0.11, 0.25] | 0.01 [-0.19, 0.21] | 1.29 [-0.77, 3.35] | 1.25 [-1.15, 3.65] |
| Thursday | 0.02 [-0.16, 0.20] | -0.03 [-0.23, 0.17] | 1.54 [-0.53, 3.61] | -0.15 [-2.56, 2.27] |
| Saturday | 0.08 [-0.11, 0.26] | -0.17 [-0.38, 0.03] | 0.62 [-1.50, 2.74] | -1.19 [-3.68, 1.31] |
| Sunday | 0.07 [-0.11, 0.26] | -0.14 [-0.34, 0.06] | -0.22 [-2.32, 1.89] | 0.00 [-2.47, 2.47] |
| *n,* # of observations | 195, 4073 | 193, 3244 | 196, 4130 | 194, 3257 |
| **Diary TST** | | | | |
| Within Lagged Outcome (fixed) | **0.17*** [0.14, 0.20],**  **0.02** | **0.10*** [0.06, 0.14],**  **0.01** | **0.20*** [0.17, 0.22],**  **0.03** | **0.15*** [0.12, 0.18],**  **0.01** |
| Within Lagged Outcome (random) | **0.02***** | **0.03***** | **--** | -- |
| School Term | 0.05 [-0.13, 0.22],  <0.01 | -0.06 [-0.27, 0.15],  <0.01 | **2.54* [0.53, 4.56],**  **<0.01** | 1.73 [-0.78, 4.24], <0.01 |
| Body Mass Index | 0.02 [-0.01, 0.06],  <0.01 | 0.02 [-0.01, 0.06],  <0.01 | -0.07 [-0.54, 0.41],  <0.01 | 0.19 [-0.32, 0.70],  <0.01 |
| Born in Australia | 0.10 [-0.25, 0.46],  <0.01 | 0.14 [-0.22, 0.49],  <0.01 | -3.01 [-7.45, 1.44],  <0.01 | -2.32 [-7.09, 2.45],  <0.01 |
| Female | 0.29 [-0.03, 0.62],  <0.01 | **0.45** [ 0.12, 0.77], 0.01** | **5.25* [1.18, 9.32],**  **0.01** | **7.18** [2.80, 11.56],**  **0.02** |
| Year Level | -0.11 [-0.33, 0.11],  <0.01 | 0.05 [-0.17, 0.27],  <0.01 | -0.18 [-2.92, 2.56],  <0.01 | 2.88 [-0.06, 5.83],  0.01 |
| Non-white | **-0.50** [-0.86, -0.14],**  **0.01** | **-0.36 [-0.72, 0.01], 0.01** | **-4.76* [-9.29, -0.23],**  **0.01** | -3.40 [-8.29, 1.48],  0.01 |
| Working | 0.03 [-0.16, 0.23],  <0.01 | 0.05 [-0.15, 0.25], <0.01 | **3.29** [0.84, 5.74],**  **0.01** | **3.40* [0.75, 6.05],**  **0.01** |
| Parental Marital Status | 0.18 [-0.23, 0.59], <0.01 | -0.21 [-0.62, 0.21],  <0.01 | 2.47 [-2.68, 7.61],  <0.01 | 0.25 [-5.35, 5.85],  <0.01 |
| Morningness Eveningness | **-0.04*** [-0.06, -0.02], 0.02** | 0.01 [-0.01, 0.03],  <0.01 | **-0.36** [-0.62, -0.10], 0.02** | 0.07 [ -0.21, 0.36], <0.01 |
| Study Day | **-0.02** [-0.03, -0.01],**  **<0.01** | -0.01 [-0.02, 0.002], <0.01 | **-0.15* [-0.27, -0.04],**  **<0.01** | -0.06 [-0.21, 0.09],  <0.01 |
| Day of the Week  (effect size) | <.001 | <.001 | <.001 | <.001 |
| Monday | 0.20* [ 0.04, 0.36] | 0.07 [-0.13, 0.26] | **2.32* [0.48, 4.16]** | 1.84 [-0.48, 4.16] |
| Tuesday | 0.07 [-0.09, 0.24] | -0.04 [-0.23, 0.16] | **0.16 [-1.75, 2.07]** | 0.11 [-2.20, 2.43] |
| Wednesday | 0.06 [-0.10, 0.23] | 0.03 [-0.16, 0.22] | 0.57 [-1.34, 2.47] | 1.26 [-1.05, 3.57] |
| Thursday | 0.01 [-0.15, 0.18] | -0.001 [-0.19, 0.19] | 1.28 [-0.63, 3.19] | 0.70 [-1.63, 3.03] |
| Saturday | 0.12 [-0.05, 0.29] | -0.07 [-0.27, 0.12] | 1.38 [-0.57, 3.33] | 0.15 [-2.24, 2.53] |
| Sunday | 0.12 [-0.05, 0.29] | -0.01 [-0.21, 0.18] | 0.53 [-1.42, 2.47] | 1.56 [-0.81, 3.92] |
| *n,* # of observations | 203, 4618 | 202, 3527 | 204, 4683 | 202, 3523 |
| **Diary SE** | | | | |
| Within Lagged Outcome (fixed) | **0.17*** [0.13, 0.20],**  **0.02** | **0.10*** [0.06, 0.14],**  **0.01***** | **0.20*** [0.17, 0.22],**  **0.03** | **0.14*** [0.11, 0.17],**  **0.01** |
| Within Lagged Outcome (random) | **0.02***** | **0.02***** | **--** | -- |
| School Term | **0.19* [ 0.02, 0.36],**  **<0.01** | 0.10 [-0.11, 0.31],  <0.01 | **4.90*** [2.88, 6.92],**  **<0.01** | **3.80** [1.30, 6.30],**  **<0.01** |
| Body Mass Index | 0.02 [-0.02, 0.06],  <0.01 | 0.02 [-0.02, 0.06],  <0.01 | -0.13 [-0.60, 0.34],  <0.01 | 0.18 [-0.34, 0.69],  <0.01 |
| Born in Australia | 0.07 [-0.27, 0.42], <0.01 | 0.10 [-0.26, 0.46],  <0.01 | -3.12 [-7.56, 1.32],  <0.01 | -2.28 [-7.10, 2.53], <0.01 |
| Female | 0.20 [-0.12, 0.51],  <0.01 | **0.40* [ 0.07, 0.73],**  **0.01** | **4.74* [0.64, 8.83],**  **0.01** | **7.16** [2.70, 11.62],**  **0.02** |
| Year Level | -0.17 [-0.38, 0.04],  <0.01 | 0.03 [-0.20, 0.25],  <0.01 | -0.68 [-3.40, 2.04],  <0.01 | **3.12* [0.15, 6.09],**  **0.01** |
| Non-white | **-0.41* [-0.76, -0.06],**  **0.01** | -0.31 [-0.67, 0.06],  0.01 | -4.14 [-8.66, 0.38],  0.01 | -3.42 [-8.37, 1.54],  0.01 |
| Working | 0.02 [-0.16, 0.21],  <0.01 | 0.05 [-0.15, 0.25],  <0.01 | **3.32** [0.91, 5.73],**  **0.01** | **3.48* [0.83, 6.13],**  **0.02** |
| Parental Marital Status | 0.08 [-0.32, 0.48],  <0.01 | -0.22 [-0.64, 0.20],  <0.01 | 1.69 [-3.42, 6.79],  <0.01 | 0.38 [-5.28, 6.03],  <0.01 |
| Morningness Eveningness | **-0.04*** [-0.06, -0.02],**  **0.03** | 0.01 [-0.02, 0.03]  <0.01 | **-0.39** [-0.65, -0.13], 0.02** | 0.06 [ -0.22, 0.35], 0.02 |
| Study Day | **-0.01** [-0.02, 0.004],**  **<0.01** | -0.01 [-0.02, 0.004], <0.01 | -0.11 [-0.23, 0.01],  <0.01 | -0.04 [-0.19, 0.12],  <0.01 |
| Day of the Week  (effect size) | <.001 | <.001 | <.001 | <.001 |
| Monday | 0.22** [ 0.06, 0.38] | 0.05 [-0.14, 0.25] | **2.86** [1.00, 4.73]** | 1.93 [-0.42, 4.28] |
| Tuesday | 0.08 [-0.08, 0.25] | -0.05 [-0.25, 0.14] | 0.44 [-1.50, 2.39] | -0.03 [-2.37, 2.32] |
| Wednesday | 0.07 [-0.09, 0.24] | 0.01 [-0.19, 0.20] | 0.84 [-1.10, 2.77] | 1.09 [-1.26, 3.43] |
| Thursday | 0.00 [-0.17, 0.16] | -0.03 [-0.23, 0.17] | 1.21 [-0.73, 3.15] | 0.37 [-1.99, 2.73] |
| Saturday | 0.05 [-0.12, 0.22] | -0.16 [-0.36, 0.04] | 0.59 [-1.38, 2.57] | -1.11 [-3.52, 1.29] |
| Sunday | 0.05 [-0.12, 0.22] | -0.09 [-0.29, 0.11] | -0.42 [-2.39, 1.55] | 0.58 [-1.80, 2.96] |
| *n,* # of observations | 202, 4588 | 201, 3496 | 203, 4649 | 202, 3520 |
| *Note*. Values presented are unstandardised coefficients [95% confidence intervals], Cohen’s *f*^2^. **p < .*05*, **p < .*01, ****p* < .001. Bold indicates a significant association. Random effects for within-person level lagged outcomes were included in all of the initial models, and subsequently dropped if the model did not converge (represented by --); Cohen’s *f*^2^ are presented for the models with random effects for the within-person predictor. For day of week, a single Cohen’s *f*^2^ effect size is presented for the covariate, before individual unstandardised coefficients and [95% confidence intervals] for each day; Tuesday was used as the reference level. | | | | |
